# Editorial: Resolving the Complexity of Plant Genomes and Transcriptomes With Long Reads

**DOI:** 10.3389/fpls.2021.832257

**Published:** 2022-01-21

**Authors:** Agnieszka Zmienko, Pawel Wojciechowski, Marek Figlerowicz

**Affiliations:** ^1^Institute of Bioorganic Chemistry of Polish Academy of Sciences, Poznan, Poland; ^2^Faculty of Computing and Telecommunications, Institute of Computing Science, Poznan University of Technology Poznan, Poznan, Poland

**Keywords:** long read DNA sequencing, alternative splicing, transcriptome, genome assembly, PacBio, nanopore

Plant genomes are extremely variable in terms of their sizes and ploidy levels. Another characteristic of plant genomic DNA is the high number of repeated sequences, including transposable elements and tandem repeat arrays. In some extreme cases, like in maize, the repetitive elements constitute as much as 80% of the genome. Additionally, extensive gene copy number variations had been observed in plants. Remarkably, the regions of the highest structural diversity turned out to contribute to a vast amount of phenotypic variation and determine traits important for breeding and agriculture. Third-generation sequencing technologies, including single-molecule real-time (SMRT) and nanopore sequencing, are capable of delivering unprecedentedly long reads, opening entirely new perspectives for structural and functional genomic studies. This Research Topic presents research findings from the fields of plant structural and functional genomics, illustrating recent progress in this area.

One of the most remarkable advances, brought about by third-generation sequencing, is improvements in the completeness and availability of eukaryotic genome assemblies. Long-read data-based assemblies provide insight into so-far poorly characterized genomic regions, e.g., centromeres, which are rich in repetitive elements and thus extremely difficult to investigate using short-read sequencing data only. Liu et al. reported assembling a highly contiguous genome for yellowhorn (*Xanthoceras sorbifolium*), which is an oil-producing tree from the Sapindaceae family, native to dryland in northern China. By combining long-read and Hi-C sequencing data, they obtained chromosome-level genome assembly, identified centromeric regions, and showed their enrichment in LINE1 and Gypsy elements. Additionally, by comparing their assembly with the available genomes of two other cultivars, they demonstrated substantial intraspecies variation of yellowhorn. Finally, after annotating the genome, they identified the candidate genes involved in the biosynthesis of nervonic acid, which is one of the very long-chain fatty acids, present in the seed oil of yellowhorn.

Using long-read data increases the possibility of assembling heterozygous genomic regions into separate contigs. This may affect the total size of the assembly, which eventually appears to be larger than expected. Duplicated gene content will also be detected in such assembly with tools that analyze its completeness by searching for single-copy orthologs, e.g., BUSCO. However, haplotype separation may be the desired outcome, since it may provide a unique insight into the allele composition in each haplotype. This has been demonstrated by Melnikova et al. who combined short- and long-read sequencing to create high-quality genomic assemblies of male and female individuals of poplar hybrid *Populus* × *sibirica*. They observed the separation of haplotypes in large parts of the assemblies, including sex-determining genomic regions (SDRs). Thanks to that, the authors were able to characterize SDR variants separately in X and Y haplotypes and suggest which ancestor species they originated from.

With the increased availability of DNA sequencing, the creation of a genomic assembly could represent an important advancement for follow-up postgenome studies. Shyamli et al. combined long- and short-read sequencing to assemble and annotate the genome of the “miracle tree” *Moringa oleifera* native to the Indian subcontinent, known for its fast growth and high medicinal and nutritional properties. In the next step, the authors generated a genome-guided transcriptome and focused on the expression analysis of the heat shock transcription factor gene family, to move closer to understanding of the genetic factors underlying *M. oleifera* drought tolerance.

The applications of third-generation sequencing are not limited to assembling genomes. For example, the ability to sequence one transcript in a single long read is a benefit for transcriptomic studies, as it enables the accurate identification and spatial analysis of alternative splicing events. Tu et al. performed hybrid RNA sequencing on samples acquired from various tissues of *Liriodendron chinense*, a deciduous tree species of the Magnoliaceae family. They analyzed the transcriptomic landscape, alternative splicing, and polyadenylation events in this species. Thanks to applying long-read technology, they identified new isoforms of known genes, but also found new genes and long non-coding RNAs, thus creating a useful resource for improving the annotation of the recently published *L. Chinense* genome. They also used the data for coexpression network analysis.

The rapid development of new long-read sequencing applications raises the need to adapt existing bioinformatic tools and to create brand new algorithms, capable of dealing with long reads and the specific information they provide. Zhang et al. addressed this issue with regard to extrachromosomal circular DNA (eccDNA) analysis. Although eccDNA has been observed in eukaryotic cells for a long time, it recently focused attention on its putative role in adaptation and cancer. Zhang et al. introduced the ecc_finder, a new algorithm that allows an efficient and accurate eccDNA detection both in short-read and long-read sequencing data. Due to a wide range of expected eccDNA sizes, the latter has more potential to span the full length of individual eccDNA in one read, thus providing comprehensive information about its sequence and the genomic locus it was derived from.

While the selection of long-read data applications presented in this Research Topic is by no means exhaustive, we believe that it accurately reflects the broadness and potential of third-generation sequencing technology, promising new exciting discoveries in all fields of plant genomics.

## Author Contributions

AZ, PW, and MF wrote the editorial. All authors contributed to the article and approved the submitted version.

## Conflict of Interest

The authors declare that the research was conducted in the absence of any commercial or financial relationships that could be construed as a potential conflict of interest.

## Publisher's Note

All claims expressed in this article are solely those of the authors and do not necessarily represent those of their affiliated organizations, or those of the publisher, the editors and the reviewers. Any product that may be evaluated in this article, or claim that may be made by its manufacturer, is not guaranteed or endorsed by the publisher.

